# Gitelman syndrome diagnosed in the first trimester of pregnancy: a case report and literature review

**DOI:** 10.1515/crpm-2021-0075

**Published:** 2022-12-19

**Authors:** Yang Cao, Dan Hu, Peng Yun, Xinwei Huang, Yan Chen, Fangping Li

**Affiliations:** Department of Endocrinology, The Seventh Affiliated Hospital, Sun Yat-sen University, Shenzhen, Guangdong, China

**Keywords:** gitelman syndrome, hypocalciuria, hypokalemia, hypomagnesemia, pregnancy

## Abstract

**Objectives:**

Gitelman syndrome is a rare salt-losing tubulopathy caused by inactivating mutations in the *SLC12A3* gene, which is expressed in the distal convoluted tubule and accounts for 5–10% of renal sodium reabsorption. Atypical symptoms and insidious conditions generally delay diagnosis until childhood or even adulthood. Here, we report the case of a 22-year-old Chinese woman who was admitted to our endocrinology department for severe hypokalemia during pregnancy.

**Case presentation:**

The patient had no specific symptoms but exhibited hypokalemia, metabolic alkalosis, hypomagnesemia, hypocalciuria, hyperreninemia, hyperaldosteronism, and normal blood pressure. Together, these symptoms indicated the clinical diagnosis of Gitelman syndrome, which was confirmed by genetic analysis. Many drugs have limited safety data during early pregnancy, and optimum potassium and magnesium levels are necessary for a successful pregnancy.

**Conclusions:**

Diagnosis and management of Gitelman syndrome are crucial during pregnancy to ensure the safety of the mother and fetus, especially during the first trimester.

## Introduction

Gitelman syndrome (GS) is a rare, autosomal recessive, inherited salt-losing tubulopathy that was first reported by Gitelman et al. in 1966 [1]. The disease is caused by biallelic inactivating mutations in the *SLC12A3* gene, which is located on chromosome 16q13 and encodes the thiazide-sensitive sodium chloride cotransporter (NCCT) expressed in the distal convoluted tubule [2]. Patients with Gitelman syndrome may be asymptomatic or have relatively mild or nonspecific symptoms, such as salt-craving, fatigue, dizziness, weakness, thirst, muscle weakness, nocturia, or cramps. Symptom onset usually occurs after the age of 6 years [3], but can be delayed until adolescence or adulthood, when diagnosis may be incidentally established during a routine blood sample test. In addition, some patients exhibit severe symptoms, such as puberty delay, chondrocalcinosis, tetany, rhabdomyolysis, seizures, and ventricular arrhythmia [4], [5], [6]. The characteristics on laboratory testing include hypokalemia, metabolic alkalosis, hypomagnesemia, hypocalciuria, and normal blood pressure, despite hyperreninemia and hyperaldosteronism. Hypomagnesemia due to renal magnesium wasting, often accompanied by hypocalciuria, develops in approximately 50% of patients with GS [7]; however, this can also appear in other salt-losing cases, such as Bartter syndrome type Ⅲ. The use of symptoms and laboratory tests to differentiate from other salt-losing nephropathies may be difficult. According to the consensus and guidance of GS in 2016, the criterion for establishing a diagnosis of GS is still genetic testing (identification of biallelic inactivating mutations in *SLC12A3*) [8]. GS may involve either an atypical symptom or an insidious clinical condition that can be easily ignored by patients and even doctors. The prevalence of GS is low; in Asia, since the prevalence is only 10.3 per 10,000 people [9], pregnancy with Gitelman syndrome is even rarer. Gitelman syndrome during pregnancy can acutely aggravate hypokalemia or other severe complications. However, diagnosis and management during pregnancy remain challenging. Herein, we report a case of a pregnant woman with Gitelman syndrome diagnosed incidentally during pregnancy testing.

## Case presentation

A 22-year-old pregnant woman, gravida 5, para 1, three miscarriages in the 5th week of gestation was admitted to the Department of Endocrinology for the evaluation of severe hypokalemia detected during pregnancy testing in the gynecology department. 16 years prior, at the age of 6 years, she began to have cramps after crying or losing her temper, without dizziness, seizures, or other symptoms. Laboratory tests revealed hypokalemia (data missing), and the symptoms were relieved after potassium supplementation. Subsequently, she experienced recurring cramps after crying, losing her temper, or fatigue. The symptoms were relieved without any treatment and had no effect on her daily life; therefore, she did not pay attention to these symptoms. Two days prior to admission, she underwent a laboratory test for pregnancy. Her blood test result showed severe electrolyte disturbances: her serum potassium level was 2.42 mmol/L and magnesium level was 0.65 mmol/L, but she had no symptoms. After intravenous and oral potassium chloride supplementation in the emergency department, the patient was admitted to the Department of Endocrinology for investigation of hypokalemia.

**Figure 1:**
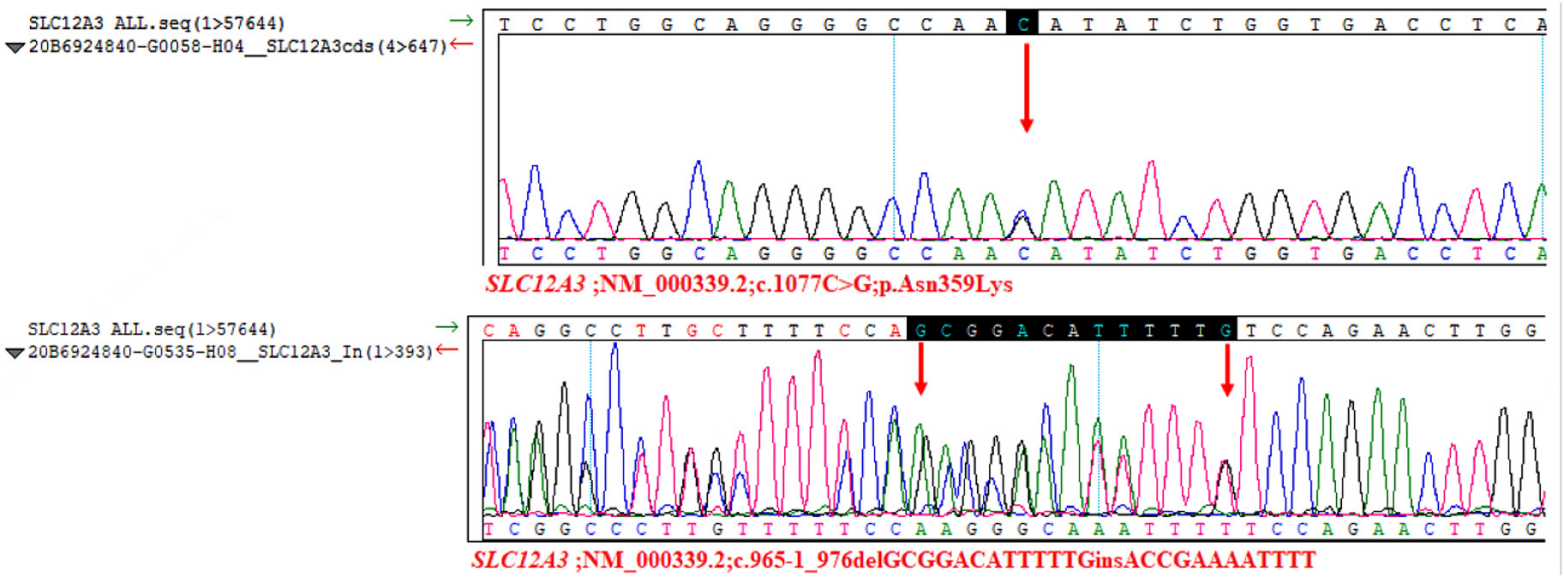
Mutation sites of *SLC12A3*.

At presentation, the patient had no sweating, vomiting, diarrhea, or anorexia. She denied laxative or diuretic abuse, hypokalemia, or cramps in other family members. Physical examination revealed a height of 149 cm, weight of 46.3 kg and blood pressure of 97/71 mmHg. There were no positive signs on physical examination, except for the Trousseau sign. Detailed laboratory examinations were performed for further diagnosis and treatment. Laboratory analysis revealed hypokalemia, metabolic alkalosis, renal potassium wasting, hypomagnesemia, hypocalciuria, hyperreninemia, and hyperaldosteronism (Table 1) with normal blood pressure, which indicated a clinical diagnosis of GS. The diagnosis was confirmed when a heterozygous mutation c.1077C>G (p.Asn359Lys) and c.965-1_976delGCGGACATTTTTGinsACCGAAAATTTT in *SLC12A3* was identified by whole exome sequencing (Figure 1). After the electrolyte levels were maintained within a relatively optimal range, the intravenous treatment was changed to oral supplementation.

**Table 1:** Blood and urine laboratory examinations.

Laboratory data	Results	Reference range
Hematology		
White blood cell	9.53 × 10^9^/L	3.50–9.50
Hemoglobin	132 g/L	115–150
Platelet	415 × 10^9^/L	125–350

Biochemistry		

Urea	2.5 mmol/L	2.6–7.5
Creatinine	47umol/L	41–73
Carbon dioxide	30 mmol/L	20–30

Arterial blood gas		

PH	7.53	7.35–7.45
PaCO_2_	32 mmHg	35–48
Bicarbonate	28.3 mmol/L	21.0–28.0
Base excess	4.4 mmol/L	−3.0∼+3.0
Sodium	133 mmol/L	136–145
Potassium	2.40 mmol/L	3.40–4.50
Chloride	97 mmol/L	98–107
Ionized calcium	1.01 mmol/L	1.05–1.27

Serum electrolyte		

Sodium	136 mmol/L	137–147
Potassium	2.70 mmol/L	3.50–5.30
Chloride	96 mmol/L	99–110
Calcium	2.29 mmol/L	2.11–2.52
Phosphate	0.96 mmol/L	0.85–1.51
Magnesium	0.55 mmol/L	0.75–1.02

Hormone		

Parathyroid hormone	67 pg/mL	15.0–68.3
Plasma renin activity	40.08 ng/mL/h	1.31–3.95
Aldosterone	781.34 pg/mL	40–310

Urine parameters		

24 h urine volume	3.86 L	1.0–2.5
24 h urine potassium	113 mmol/24 h	25–100
24 h urine sodium	262 mmol/24 h	130–260
24 h urine chlorine	300 mmol/24 h	170–250
24 h urine calcium	<1.9 mmol/24 h	2.5–7.5
24 h urine creatinine	8.65 mmol/24 h	2.90–14.10
Fractional excretion of chloride	16.97%	
Spot potassium-creatinine ratio	11.54 mmol/mmol	
Spot calcium-creatinine ratio	0.16 mmol/mmol	

## Discussion

Physiological and biochemical changes during pregnancy and at different gestational weeks have their own reference values, which may help clinicians to distinguish between physiological and pathological changes [10]. During normal pregnancy, one of the most important accommodations is by the renal system, with changes in glomerular hyperfiltration, altered tubular function, and shifted electrolyte-fluid balance [11]. Nutrients and electrolytes necessary for fetal growth accumulate because of altered tubular function. Total body sodium increases by an average of 3–4 mEq/d, and total potassium also increases by up to 320 mEq by the end of gestation [12], in which atrial natriuretic peptide, progesterone, aldosterone, deoxycorticosterone, and Na^+^/K^+^ transporters are all involved in the regulation of electrolytes. Furthermore, disorder of any aspect may exacerbate electrolyte retention or loss during this physical status.

**Figure 2:**
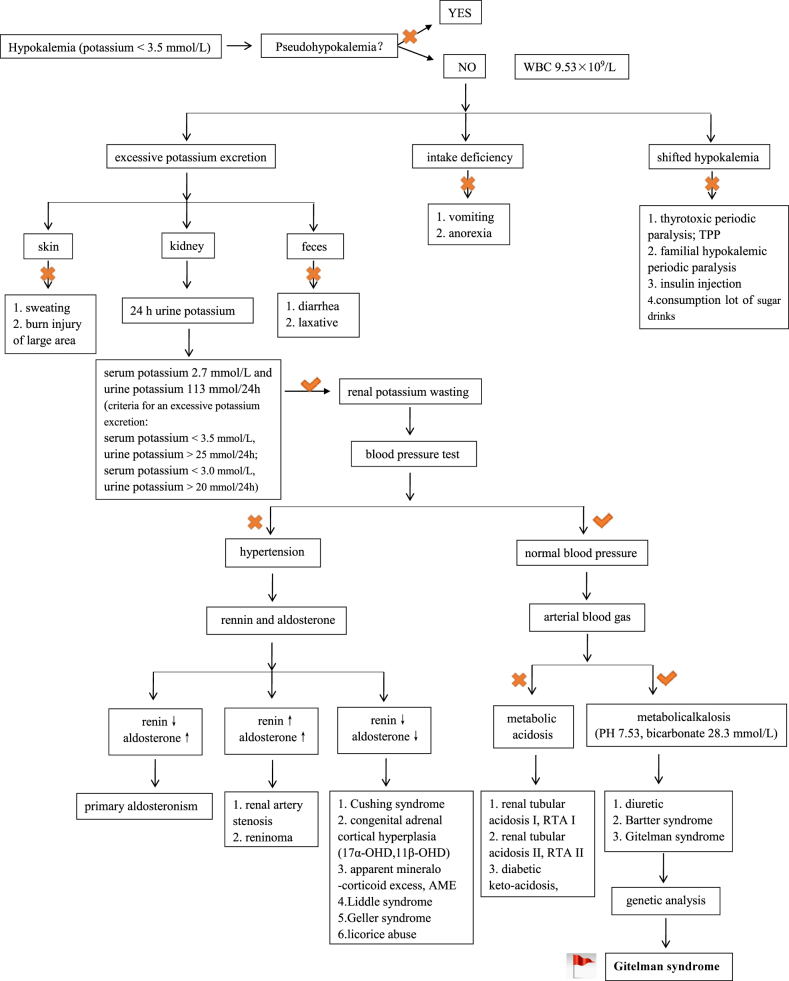
Diagnostic approach to hypokalemia.

Here, we report the case of a woman with long-term chronic hypokalemia who was incidentally diagnosed with GS during a pregnancy test. The most important diagnostic clue for this patient was not her symptoms or signs, but severe hypokalemia, which has diverse causes. To establish diagnosis, first, pseudohypokalemia was excluded because her white blood cell count was almost in the normal range. Second, potassium deficiency is known to have three main causes: (1) intake deficiency, (2) excessive potassium excretion, and (3) shifted hypokalemia. The patient had no sweating, vomiting, diarrhea, or anorexia. She denied laxative or diuretic abuse, insulin injection, or a history of hypokalemia or cramps in other family members. Further, her thyroid function was normal. This indicated that intake deficiency and shifted hypokalemia were not the likely causes. The kidney is the most important organ regulating water-electrolyte and acid-base balance and accounts for the majority of potassium excretion, which may have been the cause of the patient’s hypokalemia. Twenty-four hour measurement of urine potassium can help confirm excessive potassium excretion. Third, although many diseases are characterized by excessive potassium excretion, the patient’s normal blood pressure, metabolic alkalosis, hyperreninemia, and hyperaldosteronism enabled us to reduce the scope to Bartter syndrome and Gitelman syndrome, while identifying a higher likelihood of GS for hypomagnesemia and hypocalciuria. Finally, genetic analysis further confirmed the diagnosis of GS. Figure 2 shows the diagnostic approach for hypokalemia.

Although many cases of GS have been reported, experiences in the diagnosis and management of GS during pregnancy remains limited, especially in the Asian population. In 2020, Zhang et al. analyzed the clinical and laboratory features of female Gitelman syndrome and pregnancy outcomes in a Chinese cohort [13]. Overall, 101 patients with GS were included in their study, of whom 43 were female. Of the 43 female GS patients, 14 delivered before symptom onset without taking oral potassium and magnesium, and 12 delivered after the diagnosis of GS, and the pregnancy outcomes were generally uneventful except for one fetal fatality. They also found that pregnant patients with GS had lower potassium levels, especially in the first trimester, and required higher potassium supplementation, which was also observed in our case. In addition to the inactivating mutations of NCCT, during pregnancy in patients with GS, especially in the first trimester, nausea and vomiting are inevitable, glomerular filtration rate increases as early as 4 weeks gestation, and the effect of progesterone competing with aldosterone was impaired [14]; this further aggravates the urine potassium loss, even though estradiol may enhance NCCT density of the distal convoluted tubule, which may partly compensate the loss of potassium [15]. Even though the outcome of GS pregnancies described are generally favorable, patients with GS may also have asymptomatic or indistinguishable symptoms that may cause a delay in diagnosis and therapy, and this can be life threatening. Abdelghafour Elkoundi et al. reported a GS patient at 16 weeks of gestation with hypokalemic paralysis, which is rarely seen in GS, and was first diagnosed as Guillain-Barré syndrome. In this patient, severe hypokalemia led to fetal demise, ventricular fibrillation, and refractory cardiac arrest [16]. Oligohydramnios, one of the possible aberrations in patients with GS during pregangcy, which may be related to the use of diuretic should also be considered [17], [18], [19]. The reduction in transplacental water flow and fetal urine flow caused by maternal dehydration may explain the decrease in amniotic fluid volume [19]. Minhyeok et al. reported that a pregnant woman with GS developed hemolysis, elevated liver enzymes, and low platelet (HELLP) syndrome at 33 weeks’ gestation with the clinical features of increased blood pressure (still within normal range), creatinine level, and renal protein excretion [20]. In addition to intrauterine growth restriction, gestational diabetes mellitus, miscarriages in the first trimester, premature delivery, polyhydramnios, preeclampsia, and placental abruption have also been reported in patients with Gitelman syndrome [4, 21, 22]. Thus, timely diagnosis and intensive monitoring with individualized treatment are necessary for pregnant women with GS.

In our case, whether the three miscarriages the patient had experienced earlier were related to severe hypokalemia during early gestation is still debatable. Currently, the patient is successfully pregnant and is ready to deliver. The management of GS should be individualized according to the demands during pregnancy, but reaching the normal range is difficult. Potassium and magnesium supplementation is especially crucial during the first trimester. The consensus suggests a target of serum potassium >3 mmol/L and serum magnesium >0.6 mmol/L for GS [8], and this target is also considered adequate for successful pregnancy [13]. Potassium chloride and magnesium chloride are recommended, as chloride is the major anion lost in urine. Potassium- and magnesium-rich foods are also recommended. Potassium-sparing diuretics [23, 24], such as amiloride (class B) and eplerenone (class B), can be used in GS during pregnancy [25] when persistent, symptomatic hypokalemia with supplements is insufficient despite adherence, side effects are unacceptable, or both [8]. When the patient was discharged from our department, she took potassium chloride 15 g/d and magnesium potassium aspartate one tablets, three times a day for a total of 106.2 mg/d. Although her magnesium level could not reach the target of 0.6 mmol/L, the patient had no uncomfortable symptoms, and we suggested intensive monitoring of her electrolytes.

In summary, GS is a rare disease that is often ignored by patients and doctors owing to the lack of typical symptoms. Considering the harmful effects of long-term hypokalemia and its impact on mothers and fetuses, early diagnosis, treatment, and monitoring are important. Experiences should be further accumulated by assessing the management of different cases of GS in pregnancy to better ensure the safety of both the mother and fetus.
